# Laboratory Testing of Silica Sol Grout in Coal Measure Mudstones

**DOI:** 10.3390/ma9110940

**Published:** 2016-11-22

**Authors:** Dongjiang Pan, Nong Zhang, Zhengzheng Xie, Xiaowei Feng, Yong Kong

**Affiliations:** 1 Key Laboratory of Deep Coal Resource Mining, Ministry of Education of China, China University of Mining and Technology, Xuzhou 221116, Jiangsu, China; cumtpdj@163.com (D.P.); xiezhengzheng0327@163.com (Z.X.); fxw_mining@foxmail.com (X.F.); 2 Department of Civil and Environmental Engineering, Hong Kong University of Science and Technology, Clearwater Bay, Kowloon, Hong Kong 999077, China; ykongad@ust.hk

**Keywords:** silica sol, coal measure mudstone, grouting effectiveness, sealing and reinforcement of micropores

## Abstract

The effectiveness of silica sol grout on mudstones is reported in this paper. Using X-ray diffraction (XRD), the study investigates how the silica sol grout modifies mudstone mineralogy. Micropore sizes and mechanical properties of the mudstone before and after grouting with four different materials were determined with a surface area/porosity analyser and by uniaxial compression. Tests show that, after grouting, up to 50% of the mesopore volumes can be filled with grout, the dominant pore diameter decreases from 100 nm to 10 nm, and the sealing capacity is increased. Uniaxial compression tests of silica sol grouted samples shows that their elastic modulus is 21%–38% and their uniaxial compressive strength is 16%–54% of the non-grouted samples. Peak strain, however, is greater by 150%–270%. After grouting, the sample failure mode changes from brittle to ductile. This paper provides an experimental test of anti-seepage and strengthening properties of silica sol.

## 1. Introduction

Silica sol is an environmentally friendly nano-scale grout containing a large number of individual silica particles [1,2]. In the last twenty years, the use of silica sol in geotechnical fields has gradually been increasing in the United States, Sweden, Japan, South Korea, China and other countries. Saito [3] proposed using silica sols in gravelly soil to maintain stability during shield tunnelling. McCartney et al. [4] designed a secondary containment system using silica sol to prevent oil leakage. Hernqvist et al. [5] proposed a grout mixture including silica for hardrock tunnelling. Kim et al. [6] discovered from laboratory tests that the unconfined compressive strength of an alkaline silica sol was five times greater than that of sodium silicate. In addition, the coefficient of permeability for the silica sol was lower than the standard value of 10–5 cm/s. Butrón et al. [7] conducted tests over different time periods and in different storage environments involving fall-cone, unconfined compression, triaxial, and oedometer tests. Wang et al. [8] sealed the pores and blocked pore water and confined water in an inclined shaft in the Xiaojihan coal mine using silica sol.

The publications cited above show that the application of silica sol in geotechnical fields is still in the exploratory stages, and, to date, no studies on silica sol grouting in coal measure mudstones have been done. Poorly cemented argillaceous coal measure sedimentary rocks like mudstones, shales, and siltstones have a tendency to turn into mud when they come into contact with water, especially when the rocks are disturbed by mining operations. The creep parameters of the rocks may change and they can be more easily deformed. The ways in which coal mine roadways driven in argillaceous rocks are damaged and deformed are determined by the complexity of the stress state and the varied water conditions [9,10]. Many engineering case studies have shown that grouting is an effective way to increase the bearing capacity of the surrounding rock and an important element in preventing water-induced degradation of mudstone. The cement slurry most commonly used in underground coal mines has a minimum groutability width of 0.1 mm [11]. Coal measure mudstone is a naturally heterogeneous dual-porosity medium. The average pore diameter and the dominant pore diameter in the matrix system are both in the nanometre range, meaning that commonly used cement slurry has almost no effect on the matrix, and it is only useful for sealing fractures.

Generally, the more developed the pore space of mudstone, the larger the specific surface areas will be. The specific surface area is an important index measuring adsorption performance. Under these conditions, adsorption of water will be stronger, and weathering will occur more rapidly if micropores are not sealed. Over time, cement and other grout-type reinforcing material in the rock mass gradually disintegrate and the rock’s strength declines. Many mining engineers have recognised this problem, and, therefore, sealing and reinforcing the micropores to control deformation was identified as a fundamental problem that needed to be solved.

Taking mudstone in the Huaibei mining area (Anhui Province, China) as an example and silica sol as the grouting material, this study investigated the effect of silica sol grouting on coal measure mudstones. With the assistance of X-ray diffraction (XRD), the mineralogy of the mudstone was studied and the modification on the mudstone due to grouting was also explored. A hydraulically driven grouting system was designed to simulate the grouting procedures when operated in underground coal mines. On the basis of the modification mechanism and the hydraulically driven grouting system, the mudstone’s micropores before and after grouting were investigated by surface area and pore distribution analyser, so as to reveal the seal effect of silica sol on mudstone. Additionally, mechanical properties of mudstone before and after grouting were determined by conducting uniaxial compression testing to evaluate the reinforcement effect of silica sol on mudstone. In light of these explorations, this paper provides an experimental trial for anti-seepage and strengthening properties of silica sol for engineers.

## 2. Mudstone and Silica Sol Properties

### 2.1. Mudstone

The mudstone samples were taken from the Permian Lower Shihezi Formation, the formation that forms the floor of the 8_2_ coal seam in a mine in the Huaibei coal field, the Tongting Mine. Semiquantitative XRD (Bruker Corporation, Karlsruhe, Germany) and energy-dispersive X-ray spectroscopic analyses (Bruker Corporation, Karlsruhe, Germany) show that the samples contain about 33.9% quartz, 63.6% kaolinite, and 2.5% other minerals including some barite. The kaolinite contains a small amount of Fe_2_O_3_.

**Figure 1:**
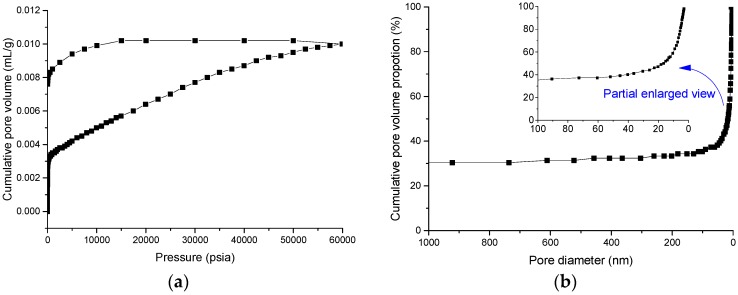
Micropore size analysis. (**a**) Graph of pore volume vs. pressure showing Hg hysteresis and retention; and (**b**) pore diameter distribution.

Tests for pore size distribution were conducted using an AutoPore IV 9510 mercury porosimeter (Micromeritics Instrument Corporation, Norcross, GA, USA). The results are shown in Figure 1. Mercury hysteresis and retention obviously occur (Figure 1a), meaning that pore structure is very complex. Figure 1b shows that more than 40% of the micropores in the sample are smaller than 20 nm in diameter.

### 2.2. Silica Sol

**Figure 2:**
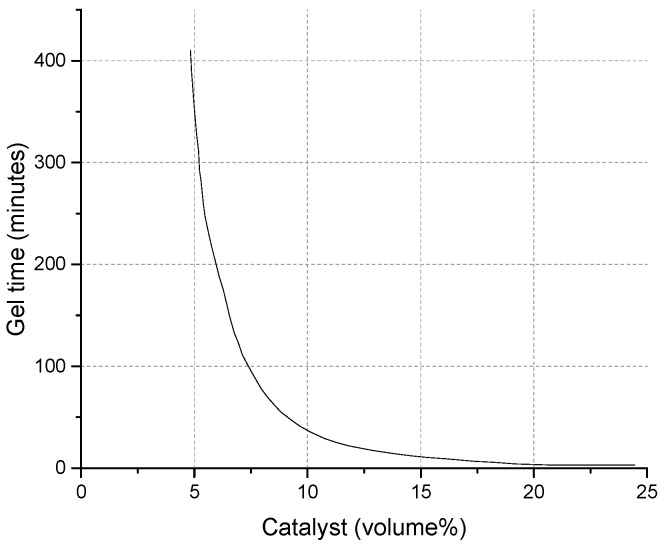
Adjustable gel time with varying catalyst dosage.

**Figure 3:**
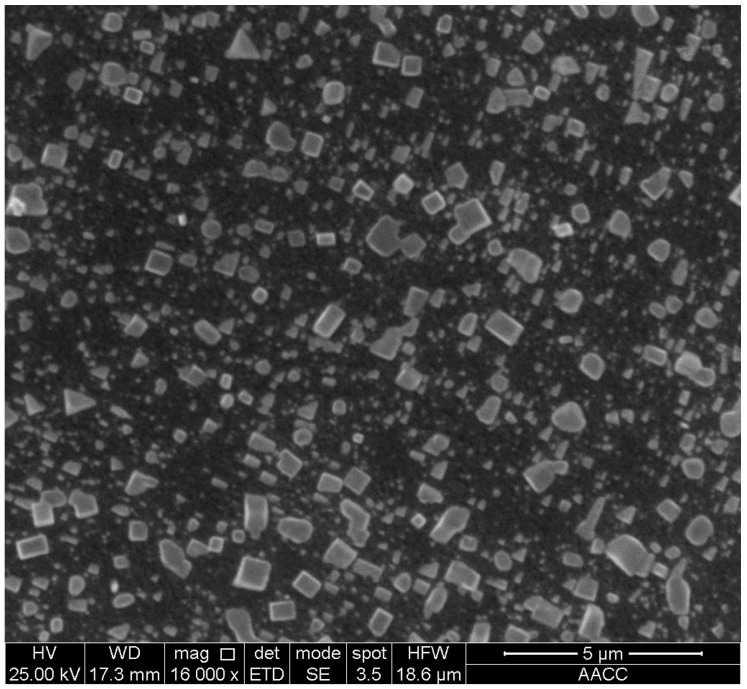
SEM backscattered electron image showing NaCl crystals distributed on the surface of hardened silica sol gel (aged 28 days). The scale bar in the lower right corner is 5 μm.

A silica sol, called MP325, was provided by BASF HOCK Mining Chemical (Jining, China) Company Limited [1,2]. Adjustable gel time with varying catalyst dosage is shown in Figure 2. The viscosity of this silica sol is 10 mPa·s and the dominant size of the silica particles is 8–12 nm. The contact angle between the silica sol and the sample was 49.26° ± 0.06° and the affinity was 47.77 mN/m, slightly lower than that for water. This means that the imbibition potential is quite significant. The catalyst is NaCl solution with a density of 1.07 kg/L and a concentration (weight %) of 10%. After the silica sol gel was hardened using a sol:catalyst ratio of 4:1, the surface properties of gel were inspected with a FEI Quanta™ 250 scanning electron microscope (Hillsboro, OR, USA). The surface of the hardened gel is smooth and covered with a layer of NaCl crystals. The structure is dense and without holes (Figure 3).

Grouting materials generally utilized in coal mines are cement (for example, P.O 425R cement) and polymer materials (for example MP364). Particle size ranges and viscosities for three grouts are shown in Table 1. P.O 425R cement and MP364 chemical high-polymer adhesive both have widespread applications in geotechnical engineering areas worldwide [1,10].

## 3. Modification Mechanism of Mudstone with Silica Sol by XRD

**Figure 4:**
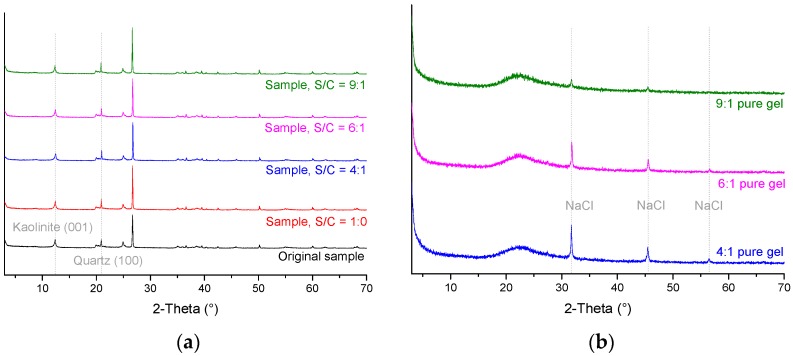
XRD results. (**a**) XRD scans showing peak positions and intensities for one untreated mudstone sample and four mudstone samples impregnated with different mixtures of silica sol grout; and (**b**) XRD test of the pure gel, the peak occurred at 20.9° or so (2-theta of quartz (100)) is too wide.

First, a mudstone sample was broken into 1–2 cm pieces (narrow samples were selected, so silica sol could be easily sucked into the samples), and the pieces were divided into five groups. Each group weighed 20 g. Second, four of the five groups were immersed in a batch of grout made from silica sol+catalyst (S/C) for 1 h, each batch having a different S/C volume ratio (the following silica sol tests all use volume ratio). Third, the four groups were placed in a drying box to cure for 2 h at 50 °C. Fourth, each of the five groups was ground into sub-325 mesh powders and a 0.5 g split was separated for XRD analysis. The XRD was done by a D8 Advance X-ray diffractometer using Cu-Kα radiation (Bruker Corporation, Karlsruhe, Germany). XRD results are shown in Figure 4a. Diffraction intensity, full width at half maximum (FWHM), and lattice spacings (calculated by the Bragg equation) for kaolinite (001) and quartz (100) are shown in Table 2. Furthermore, we conducted XRD tests on the pure gel after it was dried for 24 h at 50 °C. The pure gel could not be grinded into powder after the two-hour-drying process because of the high water content in the gel. XRD results of the pure gel are shown in Figure 4b.

Using Figure 4 and the data in Table 2 with JADE powder diffraction analysis software (Manufacturer info), it can be determined that:

1. Samples modified with silica sol maintain their original mineralogy. There are no new crystalline phases generated and the kaolinite (001) and quartz (100) lattice spacings are essentially unchanged.
2. The diffraction intensity for kaolinite (001) with pure silica sol gel increases by about 4% but decreases by 2%–17% for silica sol+catalyst. The kaolinite (001) diffraction intensity decreases with increasing S/C ratio. This suggests that the catalyst causes the kaolinite lattice to be damaged because the higher the dosage of catalyst, the more the kaolinite lattice is altered. This is because that Na^+^ in the catalyst is being exchanged with cations in the kaolinite, possibly Al^3+^. The Na^+^ may have a dispersion effect on the kaolinite making the kaolinite particles become smaller. This would result in broader and lower (001) diffraction peaks, thus an increase in FWHM.
3. Diffraction intensities for quartz (100) increase by 20%–45% after the rock is modified with silica sol. However, lower S/C ratios increase the diffraction intensity of quartz (100) more. There are a lot of reasons accounting for this phenomenon, including crystallinity, crystal size, and preferred orientation. In this test, the diffraction intensity reduction of kaolinite may be the main factor which leads to the results.

Holmboe [12] and Yu [13] used XRD to study how bentonite is modified by silica sol. Their studies found that modified bentonite retained its original mineralogy, although the (001) lattice spacing of the montmorillonite either remained unchanged or became slightly smaller. At the same time, the montmorillonite’s diffraction intensity was reduced.

## 4. Grouting Experiments

### 4.1. Design and Manufacture of a Grouting Test System

**Figure 5:**
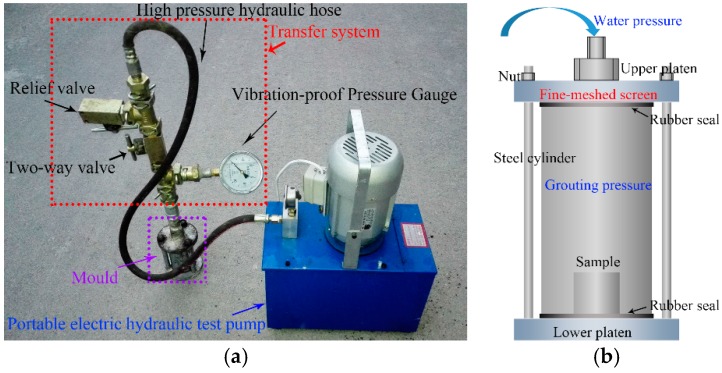
Laboratory-scale grouting system. (**a**) Grouting system; and (**b**) detail of mould assembly.

To simulate the type of grouting that is done in a mine, a laboratory-scale hydraulically driven grouting system was designed. As shown in Figure 5a, the grouting system consists of an electric hydraulic pump, a transfer system, and a mould. The pump is a reciprocating plunger pump rated at 2.5 MPa. The mould cylinder is 60 mm in diameter and 120 mm high with the upper and lower ends sealed against the upper and lower platens with rubber seals.

An important feature of the grouting system is the seals, especially the transfer system seals. A permeation grouting method is employed in many coal mines, and, with this method, a grout pressure of 1–3 MPa is commonly used. Conventional external thread type tube fittings cannot withstand that much grout pressure, so the system designed for this paper’s experiments uses K-type joints. These joints have a built-in rubber seal and a pressure rating of 60–80 MPa. A threaded connector is used between the high pressure hydraulic hose and the K-type joint, but this connector leaks. Therefore, it is necessary to clean the internal and external threads and coat them with a layer of thread locking adhesive before use.

### 4.2. Grouting Procedure

First, the rock sample is placed in the mould while the grout is being mixed for 10 s at 300–500 revolutions per min. Second, the mixed grout is dropped into the mould and three layers of fine-mesh screen are placed between the upper platen and the mould. The screens reduce damage to the sample caused by the impact of the high-pressure water.

Assembling the mould should be completed within 20–30 s because the grout hardens within a few minutes (for example, the flowing and gel times for MP364 are only 120 ± 30 s and 150 ± 30 s, respectively). The relief valve is then closed, the two-way valve opened, and the pump is turned on to pressurise the mould at 2.5 MPa for 5 min.

The last steps are to turn off the pump, release the pressure through the valves, and open the mould to remove the sample.

## 5. Sealing Tests

In an underground coal mine, sealing is the first line of defence to enable mudstones to withstand long-term deformation. Sealing with grout can improve the mudstone’s pore structure, restrict or eliminate the micro seepage channels, increase the mudstone density, and reduce its permeability. For this study, mesoporous surface area and porosity tests were conducted using a V-Sorb 2800P Surface Area and Pore Distribution Analyzer (Gold APP Instruments Corporation, Beijing, China). Because visible cracks do not affect the mesoporous analyses (mainly 2–100 nm), small, non-fractured samples were used [14].

Table 3 lists the results of the surface area and porosity tests. As shown in the table, the Brunauer–Emmett–Teller (BET) surface area of the sample after grouting with cement slurry is increased by 82.92% and total pore volume is 19.06% greater. However, the BET surface area of the sample after grouting with MP364 decreases by 46.85% and pore volume declines by 41.86% because a proportion of the pore volume is filled by the grout. The BET surface area of the sample decreases by 46.85% after grouting with silica sol if the S/C volume ratio is 6:1, but the area decreases by 61.77% if the ratio is 4:1. Similarly, the pore volumes decrease by 34.43% for the 6:1 ratio, and 52.10% for the 4:1 ratio. For silica sol, if more catalyst is used, more pore space is sealed presumably.

In summary, if cement slurry is used, the adsorption capacity of the mudstone increases and the rock’s resistance to hydration and weathering decreases. The MP364 and the MP325 silica sol both significantly decrease the adsorptive capacity of the rock. For the silica sol, the MP325 grout can fill more than 50% of the mesopore volume if the S/C ratio is as high as 4:1 using this set up. The permeability decreases with an increase in catalyst dosage. This probably occurs because the Na^+^ in the MP325 catalyst intensifies alteration of the kaolinite and some portion of the pore volume is blocked by clay minerals. A study by Qian [15] simulated sandstone with sand and cement for laboratory experiments with ZK-III chemical grout. In these tests, 9%–41% of the pore volume was filled.

**Figure 6:**
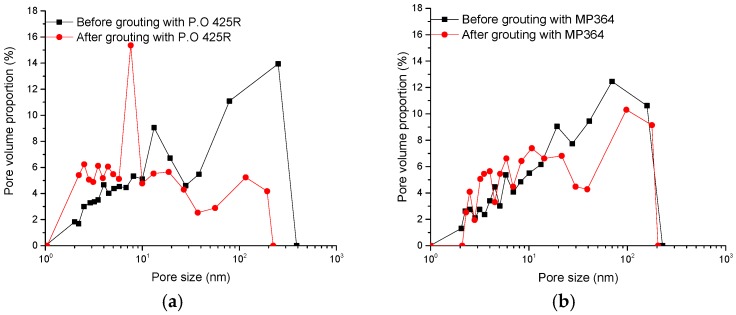

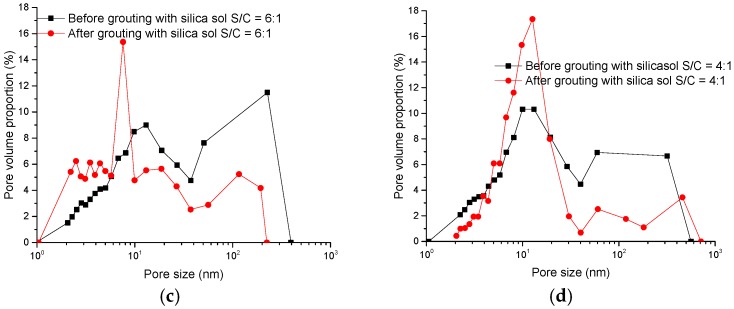
Mesopore analysis of samples before and after grouting with four different grouts. (**a**) Before and after grouting with P.O 425R; (**b**) before and after grouting with MP364; (**c**) before and after grouting with silica sol S/C = 6:1; (**d**) before and after grouting with silica sol S/C = 4:1.

Figure 6 shows plots of pore size vs. pore diameter before and after grouting for the four samples listed in Table 3. As shown in Figure 6, the dominant pore diameter decreases from more than 100 nm to about 10 nm for samples grouted with cement slurry or silica sol. The cement slurry and silica sol have a significant effect on the micro flow channels. The dominant pore diameter for the sample grouted with MP364 remains essentially unchanged, meaning that MP364 grouting does not significantly inhibit flow in the micro channels.

The general conclusion is that silica sol has the best sealing effect on micropores of the three tested grouts.

## 6. Mechanical Testing

The purpose of the tests is to show that different strengths of the grouted fracture depends on the type of grout. Because, in its natural state, the mudstone is friable and fractured, it is very difficult to prepare samples for uniaxial compression tests using standard coring techniques. For this reason, the samples used for the mechanical tests were prepared by the Langfang Branch of the Research Institute of Petroleum Exploration and Development, the research arm of the China National Petroleum Corporation. To prepare the samples, the rock was first frozen with liquid nitrogen. After the rock is solidly frozen by immersion in a closed tank of liquid nitrogen, little or no drilling fluid can permeate the frozen rock when the core sample is drilled. This means that the influence of this preparation technique on the mechanical properties of the sample is very low. During the drilling, it was found that even when attempting to drill frozen rock, it was not possible to produce standard 50 × 100 mm cores. For this reason, the cores used for the tests described below were 25 mm in diameter and 25 mm long.

**Figure 7:**
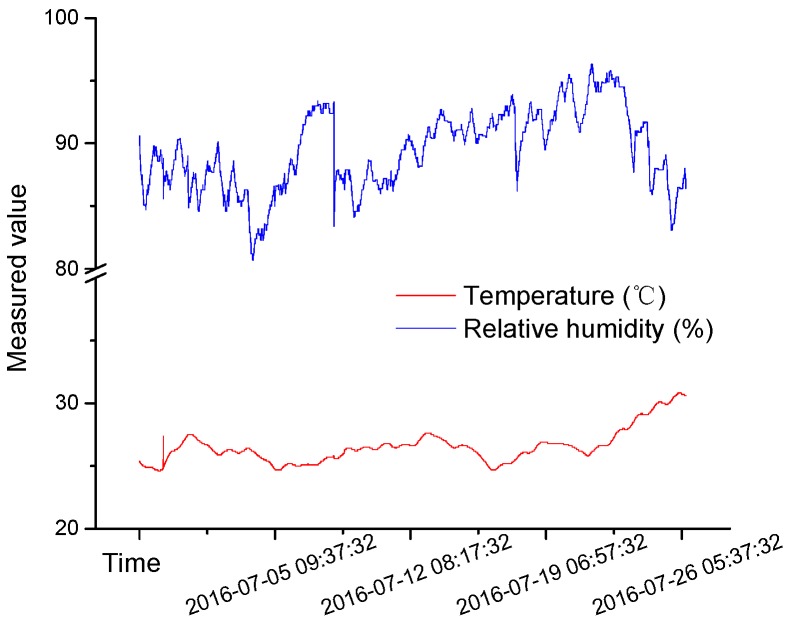
Change curves of temperature and relative humidity in the curing box.

Near-surface atmospheric changes directly affect the micro climate in the mine. Influenced by cyclic fluctuations in the temperature of the outside airflow, the air in a mine roadway also forms a relatively cyclical temperature zone with a radius of 30–40 m [16]. Owing to water evaporation, the temperature and humidity of return air, and other factors, some roadways experience high relative humidity for long periods [17]. Based on these observations, a simple sample curing box, designed to simulate temperature and relative humidity conditions in the rock surrounding a mine roadway, was constructed. The samples are placed in the curing box with a layer of water in the bottom. The layer of water ensures high relative humidity. The sample is placed on a layer of cystosepiment which covers the water. The change of temperature and relative humidity in the curing box are recorded for 28 days. The temperature is 20–30 °C, periodically changing with the atmosphere, and the relative humidity is 80%–95% (as Figure 7 shows).

**Figure 8:**
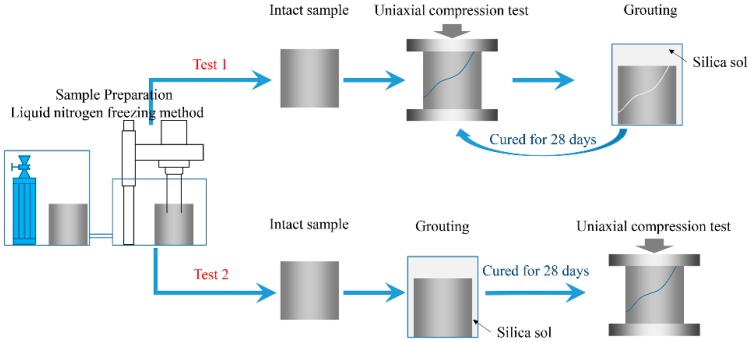
Flow chart for the uniaxial compression tests for mechanical properties.

Two different forms of uniaxial compressing testing were performed on the samples. Figure 8 shows a flow chart of the testing methods. The first style of test is to conduct uniaxial compression tests (UCT) to failure on an intact sample, grout silica sol into the fractured sample, and then conduct a UCT again after the grout has been cured for 28 days. The second style is to permeate an intact sample with a silica sol grout and then conduct UCT after curing the grout for 28 days.

When UCT was performed on intact samples for the first style of the test mentioned above, the samples were wrapped with a layer of plastic wrap. The constraint of the plastic wrap on the samples is extremely low and does not affect the UCT. The plastic wrap’s purpose was to preserve the sample’s structural integrity after the sample had ruptured at peak compression. This was necessary because the sample was to be impregnated with grout and then re-tested.

Two groups of comparison UCT were also conducted using P.O 425R and MP364. Because intact samples have very low groutability with P.O 425R and MP364, the comparison UCT was only done on fractured samples.

**Figure 9:**
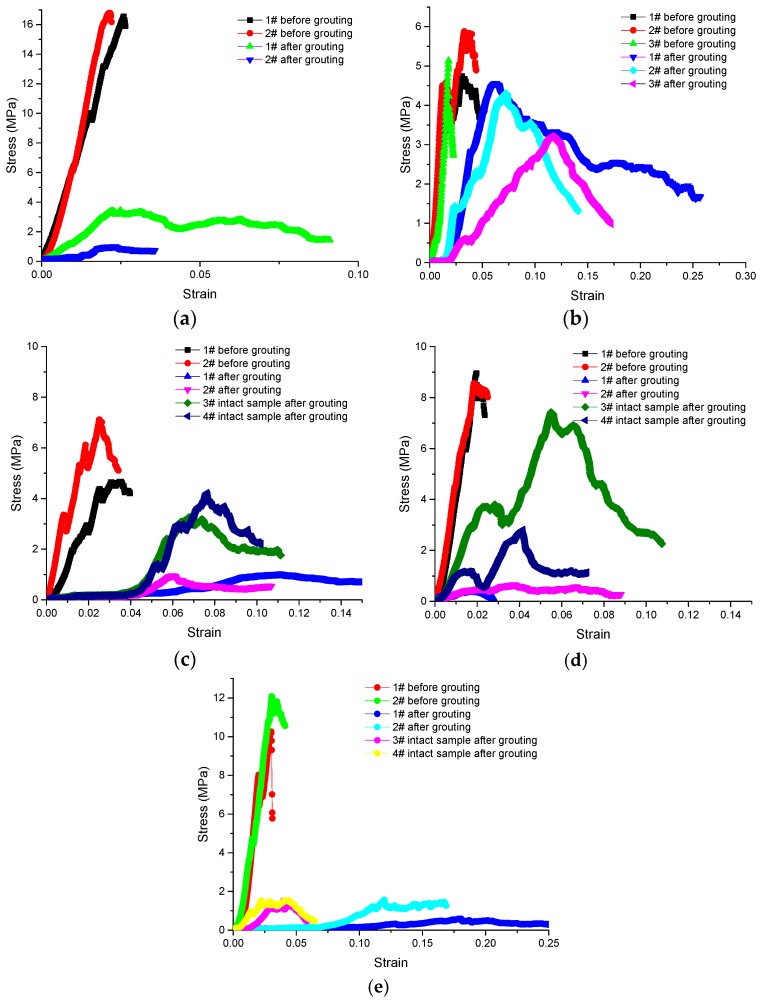
Uniaxial compression stress strain curves for samples before and after grout impregnation with five different grouts. The elastic modulus reflects the capacity of a sample to resist deformation. Uniaxial compressive strength (UCS) and peak strain both reflect the capacity of the sample to resist failure. Pre-peak absorption strain energy (PASE) [18] is the energy that causes sudden failure of a rock. (**a**) Grouting with P.O 425R W/C = 0.7; (**b**) Grouting with MP364 R/C = 1:1; (**c**) Grouting with silica sol S/C = 1:0; (**d**) Grouting with silica sol S/C = 6:1; (**e**) Grouting with silica sol S/C = 4:1.

Butrón et al. [7] showed that, after gelling, the strength of silica sol slowly rises over a six month period. To shorten the time needed for the experiments and match the time commonly used for the final strength test for cement, a 28-day curing time was chosen. The UCT were conducted using a SANS Materials Testing Machines (MTS Systems Corporation, Shenzhen, China). Test results are shown in Figure 9, Table 4 and Table 5.

To summarise, fractured samples grouted with MP364 recover their ability to resist deformation and failure very well. The brittleness of fractured samples grouted with cement slurry and silica sol decreases and the ductility is greatly improved.

Table 5 shows the results for the UCT tests done on the intact mudstone core samples after they had been grouted with silica sol. The UCT results show that, compared with the non-grouted samples, the elastic modulus of samples grouted with silica sol is 21%–38% lower and the UCS is 16%–54% less, but the peak strain is greater by factors of 1.5–2.7. The PASE is 27%–97% of the original. It is also noteworthy that after silica sol grouting, the manner in which the samples fail changes from brittle to ductile.

## 7. Conclusions

X-ray diffraction tests show that samples modified with silica sol grout maintain their original mineralogy and there are no new minerals generated. The kaolinite (001) and quartz (100) lattice spacings remain unchanged within the tested period. The intensity of the kaolinite (001) peak increases by about 4% if the rock is grouted with pure silica gel, but the peak intensity decreases by 2%–17% if the rock is grouted with silica sol and catalyst. The diffraction intensity of the peak decreases more when the grout contains more catalyst. This indicates that the catalyst damages the kaolinite lattice; a higher the proportion of catalyst in the grout means more damage to the kaolinite lattice. The intensity of the quartz (100) peak increases by 20%–45% after the sample is modified with silica sol. Decreasing the amount of catalyst used leads to increased quartz (100) peak intensity. There are a lot of reasons accounting for this phenomenon, including crystallinity, crystal size, and preferred orientation. In this test, the diffraction intensity reduction of kaolinite may be the main factor that leads to the results.

Tests on mesopores in the mudstone show that grouting with cement slurry increases the adsorption capacity of the matrix and decreases the weathering resistance and hydration resistance of the original rock. Grouting with MP364 and silica sol both significantly decrease the adsorption capacity of the mudstone matrix. The volume of mesopores filled after grouting with silica sol can be as high as 50% or more, i.e., the higher the percentage of catalyst, the more the pores are sealed presumably. The dominant pore diameter decreases from around 100 nm to about 10 nm for samples grouted with cement slurry or silica sol. The cement slurry and silica sol have a significant effect on the micro channel flow. The dominant pore diameter for samples grouted with MP364 remains essentially unchanged, meaning that MP364 does not inhibit micro channel flow. Silica sol seals micro–pores better than the other grouts used in this study.

Uniaxial compression test results from fractured samples show that fractured samples grouted with MP364 recover their ability to resist deformation and failure very well. Both UCT of rebuilt fractured samples and intact grouted samples show that, although the elastic modulus, UCS, and PASE are greatly reduced if silica sol grout is used, peak strain before failure is greater and mode of samples failure changes from brittle to ductile. Because the strength of silica sol slowly rises and does not reach final stable value over six months [7], the test results presented in this paper have a few limitations. Moreover, it was not possible to drill standard 50 × 100 mm cores from these coal measure mudstones for the UCT measurements, so the small size of the samples used means that some bias in the results is inevitable. More advanced sampling methods and instruments are needed if the results of the tests presented here are to be improved.

The ability of silica sol grout to strengthen rocks quickly is weak, but the anti-seepage performance is outstanding. Therefore, when choosing a grout for application in the field, engineers should consider both the strengthening ability and the anti-seepage performance of silica sol.

## Figures and Tables

**Table 1:** Particle size ranges and viscosities for silica sol and two grouts commonly used in geotechnical engineering areas.

Grout	Maximum Frequency Size (nm)	Viscosity (mPa·s)
MP 325	8–12	<10
P.O 425R ^1^	3.0 × 10^5^–3.5 × 10^5^	10–20
MP364 ^2^	955	250–450

^1^ P.O 425R is a quick solidification grouting cement from China United Cement Group Corporation Limited most commonly used at a water-cement mass ratio (W/C) of about 0.7. The following cement tests all use this mass ratio; ^2^ MP364 is a dense glue-like resin for fractured rock from BASF HOCK Mining Chemical (Jining, China) Company Limited that is recommended for use with a resin-catalyst mixing volume ratio (R/C) of 1:1. The following MP364 tests all use this volume ratio.

**Table 2:** Peak specific parameters and lattice spacings for kaolinite (001) and quartz (100).

Modification Method	Kaolinite (001)				Quartz (100)			
	2-Theta (°)	Intensity (Counts)	FWMH (°)	Lattice Spacing (nm)	2-Theta (°)	Intensity (Counts)	FWMH (°)	Lattice Spacing (nm)
Original sample	12.392	746	0.285	0.714	20.913	629	0.129	0.424
Sample, S/C = 1:0	12.396	776	0.265	0.713	20.913	850	0.104	0.424
Sample, S/C = 4:1	12.469	620	0.312	0.709	20.970	850	0.104	0.423
Sample, S/C = 6:1	12.431	680	0.283	0.711	20.931	758	0.109	0.424
Sample, S/C = 9:1	12.354	731	0.276	0.716	20.871	909	0.103	0.425

**Table 3:** BET surface area and total pore volume for four samples before and after grout impregnation.

Mixture Ratio	Test Time	BET Surface Area (m^2^/g)		Total Pore Volume (cm^3^/g)	
		Measured Value	Decrease Rate	Measured Value	Decrease Rate
P.O 425R, W/C = 0.7	Before grouting	0.29	−82.92%	1.17 × 10^−3^	−19.06%
	After grouting	0.53		1.39 × 10^−3^	
MP364, R/C = 1:1	Before grouting	0.33	49.51%	7.19 × 10^−4^	41.86%
	After grouting	0.16		4.18 × 10^−4^	
Silica sol, S/C = 6:1	Before grouting	3.63	46.85%	6.48 × 10^−3^	34.43%
	After grouting	1.93		4.25 × 10^−3^	
Silica sol, S/C = 4:1	Before grouting	4.10	61.77%	7.70 × 10^−3^	52.10%
	After grouting	1.57		3.69 × 10^−3^	

**Table 4:** Average test results from uniaxial compression tests of both untreated samples and samples impregnated with different kinds of grout after they had been tested to failure.

Mixture Ratio	Test Time	Average Elastic Modulus (MPa)		Average UCS (MPa)		Average Peak Strain (%)		Average PASE (10^6^ J/mm^3^)	
		Measured Value	Recovery Rate	Measured Value	Recovery Rate	Measured Value	Recovery Rate	Measured Value	Recovery Rate
P.O 425R, W/C = 0.7	Before grouting	927.54	15.31%	16.68	13.31%	2.37	101.69%	189.71	14.65%
	After grouting	142.01		2.22		2.41		27.79	
MP364, R/C = 1:1	Before routing	451.81	31.19%	5.25	84.38%	3.18	211.64%	101.46	120.92%
	After grouting	140.94		4.43		6.73		122.69	
Silica sol, S/C = 1:0	Before grouting	449.42	6.75%	5.89	16.30%	3.00	285.67%	92.59	30.71%
	After grouting	30.32		0.96		8.57		28.43	
Silica sol, S/C = 4:1	Before grouting	664.51	5.74%	11.17	9.67%	3.04	492.43%	140.47	27.32%
	After grouting	38.16		1.08		14.97		38.37	
Silica sol, S/C = 6:1	Before grouting	612.71	5.63%	8.76	5.82%	1.91	147.12%	76.96	12.63%
	After grouting	34.50		0.51		2.81		9.72	

**Table 5:** Average test results from uniaxial compression tests of intact samples impregnated with silica sol grout.

Mixture Ratio	Intact Sample	Silica Sol S/C = 1:0	Silica Sol S/C = 4:1	Silica Sol S/C = 6:1
Average elastic modulus (MPa)	621.20	161.44	131.82	231.20
Average UCS (MPa)	9.55	3.75	1.52	5.12
Average peak strain (%)	2.70	7.23	4.18	4.82
Average PASE (10^6^ J/mm^3^)	120.24	67.12	32.66	116.99
